# Vigor for *In Vitro* Culture Traits in *S. melongena* **×** *S. aethiopicum* Hybrids with Potential as Rootstocks for Eggplant

**DOI:** 10.1155/2014/702071

**Published:** 2014-01-27

**Authors:** Irene Calvo-Asensio, Jaime Prohens, Carmina Gisbert

**Affiliations:** Instituto de Conservación y Mejora de la Agrodiversidad Valenciana, Universitat Politècnica de València, Camino de Vera 14, 46022 Valencia, Spain

## Abstract

Hybrids of *Solanum melongena* and *S. aethiopicum* are of interest as rootstocks of eggplant, as they are highly vigorous and can incorporate resistance to several diseases. However, hybridization between both species is difficult. Therefore, protocols for *in vitro* culture are of great interest for their micropropagation and biotechnological breeding. We assessed the organogenesis response from leaf explants in four interspecific hybrids and in their parents testing two organogenic media: SIM-A, containing 6-benzylaminopurine and kinetin, and SIM-B, which contains thidiazuron. A higher regeneration capacity in the hybrids compared to their parents was observed. Whereas in interspecific hybrids and in one accession of *S. melongena* similar regeneration rates were observed for SIM-A and SIM-B, higher regeneration was found in the rest of genotypes when thidiazuron was used. Rooting ability in the interspecific hybrids was lower in *in vitro* micropropagated plants (35–60%) than in plants regenerated from explants (100%). The addition of indolbutiric acid (1 mg L^−1^) induced roots in nonrooted genotypes. In summary, we have adjusted *in vitro* culture conditions for regenerating and rooting *S. melongena* × *S. aethiopicum* hybrids. We have also demonstrated that these hybrids are heterotic for regeneration, which may be of interest for basic science studies.

## 1. Introduction

Common eggplant (*Solanum melongena* L.), also known as brinjal or aubergine, is an important vegetable crop widely consumed worldwide. Often, this crop displays insufficient levels of resistance to soil pests and diseases [1]. Therefore, the development of new rootstocks providing a combination of high levels of tolerance or resistance to soil stresses and high vigor can be useful for improving the yield and resilience of modern eggplant cultivars [2]. Interspecific hybrids between *S. melongena* and related species *S. incanum* L. or *S. aethiopicum* L. are highly vigorous [3, 4] and confer productive advantages for eggplant production when used as eggplant rootstocks [2].

In *S. aethiopicum*, resistance to *Fusarium oxysporum* f. sp. *melongenae*, *Ralstonia solanacearum*, and root-knot nematodes has been reported [5–11]. Furthermore, contrarily to the wild *S. incanum*, which presents high levels of glycoalkaloids [12], *S. aethiopicum* is a cultivated species known as scarlet eggplant [13] that presents low levels of *α*-solasonine and *α*-solamargine [14] and therefore presents no risk of translocation of these glycoalkaloids to the fruits. This is a very important issue, as translocation of alkaloids from the rootstock to the scion may produce undesirable results. For instance, eggplant fruits from plants grafted onto *Datura inoxia* P. Mill. accumulated scopolamine and atropine at levels high enough to cause poisoning [15]; nicotine was also obtained in tomato fruits from plants grafted onto *Nicotiana tabacum* L. [16]. Therefore, interspecific hybrids *S. melongena* × *S. aethiopicum* may be of interest, not only for increasing vigor of the scion and for conferring resistance to some important eggplant diseases, but also because they are safe from translocation of undesirable compounds from the rootstock to the scion and fruit.

Although interspecific hybrids between *S. melongena* and *S. aethiopicum* can be obtained by sexual hybridization, on many occasions, fruit set is low and fruits may be parthenocarpic or present very few seeds [7, 17]. Therefore, it would be desirable to have protocols available for efficient micropropagation. Also, the induction of regeneration from explants cultured *in vitro*, which is required for application of biotechnological techniques such as the genetic transformation [18], is of great interest for further improvement of these hybrids. Genetic transformation has been used in order to improve rootstocks in different crops [19–22].

The induction of regeneration in eggplant is achieved via organogenesis [23–28] or embryogenesis [25, 26, 29, 30] and some interesting traits related to abiotic [31, 32] and biotic [33, 34] stresses have been introduced by genetic transformation. Although eggplant tissues showed a high morphogenetic potential some drawbacks were found in different culture conditions, mainly, buds which fail to develop into shoots [23, 24, 27] and shoots which fail to develop roots [26, 27]. Research in *in vitro* culture of both *S. aethiopicum* and interspecific hybrids between *S. melongena* and *S. aethiopicum* is scarce [7, 35]. In *S. aethiopicum,* regeneration from cotyledons and leaf explants was described in Gisbert et al. [35] and, as what occurred in other species, it was genotype dependent. On the other hand, Collonnier et al. [7] reported regeneration of plants from calli resulting from protoplast fusions between *S. melongena* and *S. aethiopicum*.

The aim of this study is to evaluate the organogenic response of four *S. melongena* × *S. aethiopicum* hybrids which present potential interest as rootstocks, as well as their ability for rooting, which is a prerequisite for micropropagation. It was also to compare their organogenic response with that of their parents. For organogenesis induction, we have tested two shoot induction media (SIM) which differ in the growth regulators. The effect of genotype and culture medium on bud induction, shoot elongation, and the subsequent development of roots is examined.

## 2. Material and Methods

**Figure 1:**
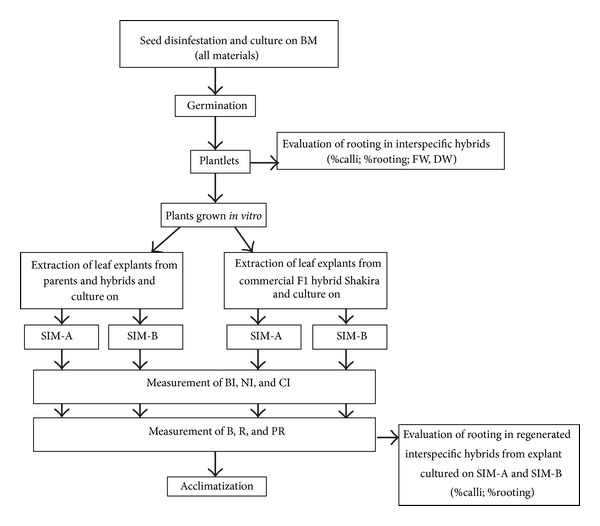
Scheme of the procedure followed for the study of the organogenic response and rooting ability of the *S. melongena*, *S. aethiopicum*, and interspecific hybrid materials.

A schema of all the assays and parameters noted along the work of our study is shown in Figure 1.

### 2.1. Plant Material, Germination, and Culture Conditions

Materials consisted of two accessions of *S. melongena *(coded as M1 and M2), two accessions of *S. aethiopicum* (coded as A1 and A2), and the four *S. melongena *×* S. aethiopicum *interspecific hybrids (Table 1). All seeds of the M1, M2, A1, and A2 parents as well as those of the interspecific hybrids were provided by Dr. John R. Stommel (ARS-USDA, Beltsville, MD, USA). Seeds came from the same harvest and were conserved under the same conditions (stored in a no-frost refrigerator at 4°C).

Seeds were surface-sterilized by immersion for 15 min in a solution of 25% commercial bleach (containing 40 g L^−1^ of active chloride) followed by three rinses in sterile distilled water and cultured in plastic Petri dishes (90 × 25 mm) sealed with parafilm containing 40 mL of basal medium (BM), which consisted of Murashige and Skoog salts [36] including vitamins (DUCHEFA, Haarlem, The Netherlands), 1.5% sucrose, and 0.7% plant agar (Figure 1). The root system of germinated plantlets and the ends of cotyledons were cut and removed, and shoots were transferred to tubes (15 cm in length and 22 mm in diameter) containing 15 mL of BM medium for rooting.

After 30 days of culture, the percentage of rooting, callus formation (presence/absence), and fresh weight (FW) and dry weigh (DW) of interspecific hybrids roots was noted in 10 plants of each genotype. DW of roots was obtained after oven drying at 70°C for 24 h.

All genotypes were micropropagated and maintained in *in vitro* culture by transferring nodes every 3-4 weeks to fresh BM. These plants were used as source of explants.

The pH of all the media was adjusted to 5.8 before sterilization at 121°C for 20 min, and cultures were incubated in a growth chamber at 26°C ± 2°C under a 16 h photoperiod with cool white light provided by Sylvania cool white F37T8/CW fluorescent lamps (90 *μ*mol m^2^ s^−1^).

### 2.2. Organogenesis Induction

Leaf explants (0.6–0.8 cm^2^) were obtained from *in vitro* cultured plants and placed with the abaxial side in contact with the shoot induction media (SIM) containing Murashige and Skoog's salts [36], 3% sucrose, and 0.7% plant agar supplemented with either 2 mg L^−1^ BA plus 0.5 mg L^−1^ Kin (SIM-A) or 0.05 mg L^−1^ TDZ (SIM-B). Growth regulators were filtered (0.22 *μ*m Millipore filters) and then added to sterilized medium. The media were plated in Petri dishes (90 × 25 mm) with 40 mL of culture medium per plate. For each combination of genotype and treatment, five repetitions (plates), with five explants per plate, were evaluated.

After 20 days of culture on SIM media, the induction of buds, necrosis appearance, and callus formation was visually assessed using these three indexes: bud index (BI), necrosis index (NI), and callus index (CI) in a scale from 0 (absence) to 4 (throughout the explant). Subsequently, the explants were transferred to BM supplemented with GA_3_ (1 mg L^−1^) for elongation. Explants producing many buds were divided into portions and tagged in order to trace their origin. The frequency of explants with organogenic buds (B), frequency of shoot regeneration (R), and mean number of shoots per explant (PR) were measured 20 days after the explants had been transferred to the elongation medium (i.e., 40 days since the beginning of cultivation of the explants in SIM media) (Figure 1).

Ten shoots isolated from regenerating explants (cultivated in either SIM-A or SIM-B and subsequently cultivated in elongation media) were transferred to tubes with BM for rooting (Figure 1). At 40 days of culture percentage of rooting was note and those without roots were subcultured to BM supplemented with indolbutiric acid (IBA) at 1 mg L^−1^ in order to induce rooting.

### 2.3. Acclimatization

A random sample of rooted plants including all tested genotypes was used for acclimatization under standard procedures. Plants were grown in a culture chamber (16 light at 25°C and 8 h dark at 23°C) in 20 mL pots filled with a mixture of peat-vermiculite (75 : 25 v : v). During the first week, each plant was protected from dehydration by covering it with an inverted transparent plastic vessel.

### 2.4. Statistical Analysis

Data was subjected to factorial analysis of variance (ANOVA). For mean separation, the Duncan multiple-range test was used. The Statgraphics Centurion XVI software (StatPoint Technologies, Warrenton, VA, USA) was used for the statistical analyses.

## 3. Results and Discussion

Interspecific hybrids between *S. melongena* and *S. aethiopicum* are of interest as rootstocks for improving the yield of eggplant [2]. These hybrids are heterotic for plant vigor, can incorporate resistance to several diseases from the *S. aethiopicum* parent, have a good grafting compatibility with eggplant, and, given their low levels of glycoalkaloids, do not present problems associated with the possible translocation of these compounds, which may be harmful for human health [3, 7, 10, 11, 14, 17]. However, up to now, no protocols for *in vitro* culture and regeneration, which are necessary for multiple biotechnological improvements, have been established for this new innovation in the field of eggplant grafting. Micropropagation, somaclonal variation, and genetic transformation are some of the techniques that can benefit from the development of regeneration protocols [18].

This study was initiated with the establishment *in vitro *and micropropagation of four interspecific *S. melongena* × *S. aethiopicum* genotypes and their respective parents. These plants were used as source of leaf explants that were cultured onto two shoot induction media (SIM): the first (SIM-A) is supplemented with 6-benzylaminopurine (BA) (the most common growth regulator used in eggplant) plus kinetin (Kin), a combination which resulted in high frequency of developed shoots per explant in the work published by Shivaraj and Rao [37]; the second (SIM-B) is supplemented with thidiazuron (TDZ), which is a good inducer of shoot regeneration in several species including eggplant and *S. aethiopicum* [26, 35].

**Figure 2:**
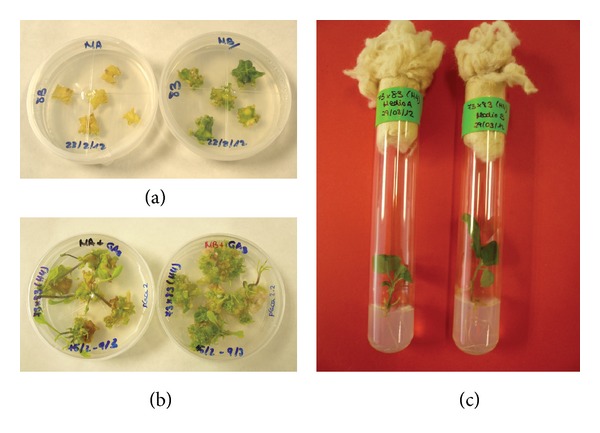
(a) Leaf explants of *S. aethiopicum* A1 at 20 days of culture on SIM-A (no response) or SIM-B (with buds). (b) Organogenic explants of the interspecific hybrid *S. melongena* × *S. aethiopicum* (M2 × A1) after 15 days of culture in the elongation medium. (c) Shoots of M2 × A1 isolated from explants cultured on SIM-A or SIM-B rooted in MB medium (at 40 days of culture).

After 20 days of culture, bud induction was observed in all the treatments with the exception of explants from genotype A2 cultured on SIM-A (Table 2). ANOVA revealed significant effects of genotype and medium for BI, NI, and CI, as well as a significant genotype × medium interaction for NI (Table 2). Among genotypes, the lowest BI values were obtained in *S. aethiopicum* genotypes (A1 and A2). In general, a higher proportion of explants with buds were obtained in SIM-B than in SIM-A, with average values of 2.27 and 1.05, respectively (Table 2). Among explants from parent (M1, M2, and A1) or hybrid (M1 × A1, M1 × A2, M2 × A1, and M2 × A2) genotypes, none or small differences were obtained for BI in SIM-B. However, in SIM-A a higher response was obtained in explants from hybrids compared to those from parents. Necrosis was present in many explants and it was higher in medium SIM-A than in medium SIM-B (Table 2, Figures 2(a) and 2(b)). The lowest levels of necrosis (NI < 1) were observed in explants of *S. melongena* M1 and M2 and in the hybrids derived from M1 (M1 × A1 and M1 × A2) cultured on SIM-B. Differences for CI were also observed among genotypes. Among parents, A1 cultured on SIM-A medium showed the lowest callus formation. In hybrids, the lowest CI values were obtained in M2 × A1 cultured on SIM-A medium (Table 2).

B, R, and PR were measured 20 days after transferring explants to the elongation medium (Table 3). ANOVA revealed significant effects of genotype and medium for B, R, and PR, as well as a significant genotype × medium interaction for the three indexes. The highest percentages of explants with buds (B = 100) were obtained in the interspecific hybrids previously induced in either SIM-A or SIM-B. The lowest values were obtained in *S. aethiopicum* genotypes A1 (B = 58) and A2, which was excluded from this analysis due to the low percentage of responding explants (BI = 0.36 on SIM-B and BI = 0.00 on SIM-A). As for the frequency of explants with organogenic buds, B was higher in SIM-B than in SIM-A. The lowest B values were obtained in explants of M2 (60%) and A1 (16%) cultured on SIM-A. Thus, TDZ resulted in a higher bud induction than BA combined with Kin (Table 3, Figure 2(a)) as it is observed with BI (Table 2). The frequency of shoot regeneration (R) was, in general, lower than B and higher in explants from hybrids than in explants from parents. In genotype A2, no explants produced shoots in SIM-A medium (Figure 2(a)). PR greatly differed among parents and hybrids (Table 3). Whereas a few number of explants regenerated more than 1 shoot per explant in parents (average PR > 1 values were only obtained in M1 and M2 cultured on SIM-A and SIM-B media, resp.), values of PR between 2 and 9 were obtained in interspecific hybrids (Table 3). The highest PR value (8.68) was obtained in M2 × A1 cultured on SIM-B. In some explants previously cultured on SIM-B vitrification was observed (Figure 2(b)). Despite this, PR values indicate that the number of healthy developed isolable shoots from explants was either similar in both media or higher in SIM-B than in SIM-A.

The results obtained for the BI and B (80% to 100%) are in agreement with the reported great morphogenetic potential of eggplant tissues for responding to organogenesis [23–28]. However, the accessions of *S. aethiopicum* (A1 and A2) could be considered of low potential or even recalcitrant for regeneration (A2). These accessions had lower organogenesis response than other *S. aethiopicum* genotypes (BBS107 and BBS116) assayed in a medium similar to SIM-B [35]. Regarding the interspecific hybrids *S. melongena* × *S. aethiopicum*, regeneration was higher than in their respective parents as it is reflected for B, R, and PR values. Thus, they are heterotic for regeneration. This effect was also observed in hybrids of *S. lycopersicum* L. × *S. pennellii* Correll, in which populations derived from this cross allowed the detection of six QTLs involved in the regeneration capacity [38]. Although in *S. melongena* the genetics of regeneration has not been studied, several genes are probably implicated.

In general, higher BI, B, R, and PR were obtained in SIM-B compared to SIM-A. Thus, at the concentrations used in our work, TDZ (SIM-B) was more effective than BA plus Kin (SIM-A) in inducing adventitious shoot regeneration from leaf explants. The effectiveness of TDZ in inducing adventitious shoot regeneration with respect to other growth regulators has been reported in several works [39–43].

Necrosis of explants and callus formation are observed in explants cultured in both SIM-A and SIM-B media, with higher NI in SIM-A and higher CI in SIM-B. Although necrosis may reduce regeneration, genotypes with similar NI have showed different regeneration response. Thus, similar NI values were observed on SIM-A cultured explants of the low responding genotype A1 and in other high responding genotypes like M1, M1 × A1, or M2 × A1. Necrosis may be related to ethylene production or accumulation in *in vitro *culture conditions [44–46]. The higher callus formation in explants cultured on SIM-B does not make the isolation of shoots difficult. Although some shoots were vitrified in this medium, similar or higher PR was observed in SIM-B versus SIM-A. It is interesting to take into account that callus formation may increase the appearance of somaclonal variation, which may be an advantage or a drawback depending on the goal of regeneration. Thus, in the case of interspecific hybrids and genotype M1, which had high regeneration in both media, depending on the aim of the induction of regeneration, the more adequate one of these two SIM can be used.

A successful rooting of *in vitro* cultured plants is a prerequisite for micropropagation or transference to field conditions [18]. In eggplant, fail in rooting has been previously reported [25–27, 37]. This problem is also commonly reported in other species in shoot induction media containing TDZ [39–43]. Thus, the rooting ability of interspecific hybrids was tested, first in the starting micropropagated plants and, secondly, in plants regenerated from leaf explants.

Roots of plantlets were excised and shoots were transferred to individual tubes containing BM. At 30 days of culture, 100% of rooting was observed in the four tested genotypes although callus formation at the base of the plants was present in some plants. The presence of calli differed among genotypes: it is observed in 37.5% of plantlets of interspecific hybrid M1 × A1, in 6.7% of M1 × A2, and in 4% of M2 × A2 (Table 4). FW and DW of roots (without calli) were measured in order to quantify putative differences for root development in the micropropagated interspecific hybrids. Among tested genotypes, a greater root development was observed in M1 × A1 when compared to the other interspecific hybrids (Table 4).

In plants isolated from explants, the frequency of rooting after 30 days of culture in MB (Table 5) was lower than that obtained in micropropagated plants (in the range between 10% and 70% versus 100%). Thus, the cytokinin used in both SIM media may be diminishing rooting capacity. Callus formation was also appreciated at the base of the shoots in all treatments although with low frequencies (between 10% and 20% in six out of the eight treatments). The differences for rooting and callus formation among genotypes could be due to different concentrations or profiles of endogenous growth regulators [40, 41]. Regarding differences from the origin of shoots, similar or higher rooting frequencies were observed in shoots from SIM-B when compared to SIM-A (Table 5). The transfer of shoots without roots to BM medium supplemented with IBA at 1 mg L^−1^ induced roots in few days. Thus, rooting is not a limiting step for regeneration of the genotypes assayed.

Standard acclimatization procedures were applied to regenerated plants with a 95% of survival.

Overall, results show that TDZ, at the low concentration used in our work (medium SIM-B), is adequate to induce buds in all tested genotypes. These buds develop into shoots able to root on BM or BM supplemented with IBA. Thus, a protocol has been established for the suitable regeneration of *S. melongena* × *S. aethiopicum* interspecific hybrids and their parents. We have also demonstrated that these interspecific hybrids are heterotic for regeneration, which may be of interest for basic science studies.

## 4. Conclusions

Taking into account all the results obtained it is concluded that a higher capacity for regeneration is observed in interspecific hybrids compared to their parents. Thus, hybrid vigor is manifested for *in vitro* culture traits. Complementation of parental genes positively influencing regeneration may be taking place in these hybrids. SIM-B seems to be better than SIM-A for bud induction as frequencies of 100% or near 100% were obtained in all tested genotypes. Development of buds into shoots was also higher in SIM-B versus SIM-A, as regenerated shoots were isolated in all genotypes. Elongation of shoots from SIM-A medium may be better, although the higher amount of buds in SIM-B medium cultured explants gives a higher proportion of isolated shoots. Although necrosis is visible in some explants and may contribute to the lack of development of buds into shoots, genotype has a great influence as genotypes with similar NI differ in R. Although rooting may fail in the regenerated shoots due to cytokinins type and/or concentration, those used in our work do not limit rooting capacity of the *S. melongena* × *S. aethiopicum* interspecific hybrids. The results are of interest for the development of *S. melongena* × *S. aethiopicum* interspecific hybrids as rootstocks for eggplant.

## Figures and Tables

**Table 1:** Plant materials used, codes, fruit characteristics, and their origins.

Plant material	Code	Fruit type	Origin
*S. melongena *			
PI263727	M1	Semilong, purple	Puerto Rico
PI470273	M2	Semilong, purple	Kalimatan, Indonesia
*S. aethiopicum *			
PI413783	A1	Very flattened, green	Burkina Faso
PI413784	A2	Very flattened, green	Burkina Faso
*S. melongena *×* S. aethiopicum *			
PI263727 × PI413783	M1 × A1	Flattened, green	Interspecific hybrid
PI263727 × PI413784	M1 × A2	Flattened, green	Interspecific hybrid
PI470273 × PI413783	M2 × A1	Flattened, green	Interspecific hybrid
PI470273 × PI413784	M2 × A2	Flattened, green	Interspecific hybrid

**Table 2:** Effect of genotype, culture medium, and their interaction on the bud index (BI), necrosis index (NI), and callus index (CI) after 20 days of culture in shoot induction media (SIM-A or SIM-B).

Factor	BI^a^	NI	CI
Genotype			
M1	1.68 c	1.42 ab	1.50 c
M2	1.72 c	1.78 bc	1.48 c
A1	1.18 b	2.52 d	0.88 ab
A2	0.18 a	2.29 cd	1.30 bc
M1 × A1	2.16 cd	2.02 bcd	0.92 ab
M1 × A2	2.46 d	1.10 a	1.24 bc
M2 × A1	2.10 cd	2.52 d	0.50 a
M2 × A2	1.82 c	1.84 bc	0.78 ab
Medium			
SIM-A	1.05 a	2.77 b	0.94 a
SIM-B	2.27 b	1.10 a	1.21 b
Genotype × medium interaction			
M1-A	0.84	2.8 de	1.64 de
M1-B	2.52	0.04 a	1.36 cde
M2-A	0.92	2.96 e	1.20 bcde
M2-B	2.52	0.60 a	1.76 e
A1-A	0.32	3.16 e	0.64 ab
A1-B	2.04	1.88 c	1.12 bcde
A2-A	0.00	3.16 e	0.96 cbd
A2-B	0.36	1.42 bc	1.64 de
M1 × A1-A	1.56	3.36 e	0.92 bc
M1 × A1-B	2.76	0.68 ab	0.92 bc
M1 × A2-A	1.88	1.52 c	1.04 bcd
M1 × A2-B	3.04	0.68 ab	1.44 cde
M2 × A1-A	1.52	3.20 e	0.20 a
M2 × A1-B	2.68	1.84 c	0.80 abc
M2 × A2-A	1.40	2.04 cd	0.92 bc
M2 × A2-B	2.24	1.64 c	0.64 ab
ANOVA^b^			
Genotype	∗∗∗	∗∗∗	∗∗∗
Medium	∗∗∗	∗∗∗	∗
Genotype × medium	ns	∗∗	ns

^a^For each of the genotype, medium, and genotype × medium interaction factors, mean values within a column separated by different letters are significantly different (*P* < 0.05) according to Duncan's multiple range test.

^b^∗∗∗, ∗∗, ∗, and ns indicate being significant, at *P* < 0.001, *P* < 0.01, and *P* < 0.05, and nonsignificant, respectively.

**Table 3:** Effect of genotype, culture medium, and their interaction on the percentage of explants with buds (B), percentage of explants with shoots (R), and number of shoots per explant with shoots (PR) after culture for 20 days in BM of leaf explants previously cultured for 20 days in shoot induction media (SIM-A or SIM-B).

Factor	B^a^	R	PR
Genotype			
M1	88 bc	54.5 b	1.11 a
M2	80 b	26.0 a	0.62 a
A1	58 a	30.0 a	0.50a
A2	nd^b^	nd	nd
M1 × A1	100 c	76.0 c	3.40 b
M1 × A2	100 c	94.0 c	5.95 c
M2 × A1	100 c	82.0 c	5.98 c
M2 × A2	100 c	78.0 c	3.22 b
Medium			
SIM-A	79.4 a	53.9 a	2.14 a
SIM-B	99.4 b	72.0 b	3.80 b
Interaction			
M1-A	80 c	65 bc	1.70 abc
M1-B	96 c	44 b	0.52 ab
M2-A	60 b	8 a	0.08 a
M2-B	100 c	44 b	1.16 abc
A1-A	16 a	0 a	0.00 a
A1-B	100 c	60 bc	1.00 abc
A2-A	nd	nd	nd
A2-B	nd	nd	nd
M1 × A1-A	100 c	68 bcd	2.04 abcd
M1 × A1-B	100 c	84 cd	4.76 def
M1 × A2-A	100 c	88 cd	5.16 ef
M1 × A2-B	100 c	100 d	6.75 fg
M2 × A1-A	100 c	76 bcd	3.28 bcde
M2 × A1-B	100 c	88 cd	8.68 g
M2 × A2-A	100 c	72 bcd	2.72 abcde
M2 × A2-B	100 c	84 cd	3.72 cde
ANOVA^c^			
Genotype	∗∗∗	∗∗∗	∗∗∗
Medium	∗∗∗	∗∗	∗∗
Genotype × medium	∗∗∗	∗	∗

^a^For each of the genotype, medium, and genotype × medium interaction factors, mean values within a column separated by different letters are significantly different (*P* < 0.05) according to Duncan's multiple range test.

^b^Not determined.

^c^∗∗∗, ∗∗, ∗, and ns indicate being significant, at *P* < 0.001, *P* < 0.01, and *P* < 0.05, and nonsignificant, respectively.

**Table 4:** Rooting ability of *S. melongena *×*  S. aethiopicum* interspecific hybrid plantlets in basal medium (MB) measured as frequency of rooting, frequency of plants with calli, and fresh weight (FW) and dry weight (DW) at 30 days of culture.

Interspecific hybrid	Plants with calli (%)^a^	Rooting (%)	FW (g)	DW (g)
M1 × A1	37.50 c	100 a	0.22 c	0.015 b
M1 × A2	6.66 b	100 a	0.15 b	0.013 ab
M2 × A1	0.00 a	100 a	0.11 a	0.008 a
M2 × A2	4.00 b	100 a	0.12 ab	0.010 a
ANOVA^b^				
Genotype	∗∗∗	ns	∗∗∗	∗

^a^Mean values within a column separated by different letters are significantly different (*P* < 0.05) according to Duncan's multiple range test.

^b^∗∗∗, ∗∗, ∗, and ns indicate being significant, at *P* < 0.001, *P* < 0.01, and *P* < 0.05, and nonsignificant, respectively.

**Table 5:** Influence of the SIM on adventitious shoot rooting. Frequency of plants with roots and basal callus formation at 30 days of culture in BM.

Factor	Rooting (%)^a^	Callusing (%)
Genotype		
M1 × A1	35 a	35 b
M1 × A2	40 a	15 a
M2 × A1	60 b	15 a
M2 × A2	35 a	40 b
Medium		
SIM-A	35 a	35.0 b
SIM-B	50 b	17.5 a
Interaction		
M1 × A1-A	10 a	50 b
M1 × A1-B	60 c	20 a
M1 × A2-A	30 ab	10 a
M1 × A2-B	50 bc	20 a
M2 × A1-A	50 bc	20 a
M2 × A1-B	70 c	10 a
M2 × A2-A	50 bc	60 b
M2 × A2-B	20 a	20 a
ANOVA^b^		
Genotype	∗	∗
Medium	∗	∗∗
Genotype × medium	∗∗	∗

^a^For each of the genotype, medium, and genotype × medium interaction factors, mean values within a column separated by different letters are significantly different (*P* < 0.05) according to Duncan's multiple range test.

^b^∗∗∗, ∗∗, ∗, and ns indicate being significant, at *P* < 0.001, *P* < 0.01, and *P* < 0.05, and nonsignificant, respectively.

## Abbreviations

BA: 6-Benzylaminopurine
BM: Basal medium
GA_3_: Gibberellic acid
Kin: Kinetin
TDZ: Thidiazuron.

## References

[1] Daunay MC, Prohens J, Nuez F (2008). Eggplant. *Handbook of Plant Breeding: Vegetables II*.

[2] Gisbert C, Prohens J, Raigón MD, Stommel JR, Nuez F (2011). Eggplant relatives as sources of variation for developing new rootstocks: effects of grafting on eggplant yield and fruit apparent quality and composition. *Scientia Horticulturae*.

[3] Prohens J, Plazas M, Raigón MD, Seguí-Simarro JM, Stommel JR, Vilanova S (2012). Characterization of interspecific hybrids and first backcross generations from crosses between two cultivated eggplants (*Solanum melongena* and *S. aethiopicum* Kumba group) and implications for eggplant breeding. *Euphytica*.

[4] Prohens J, Whitaker BD, Plazas M (2013). Genetic diversity in morphological characters and phenolic acids content resulting from an interspecific cross between eggplant (*Solanum melongena*) and its wild ancestor (*S. incanum*). *Annals of Applied Biology*.

[5] Hébert Y (1985). Comparative resistance of nine species of the genes *Solanum* to bacterial wilt *Psedomonas solanacearum*) and the nematode *Meloidogyne incognita*. Implications for the breeding of aubergine (*S. melongena*) in the humid tropical zone. *Agronomie*.

[6] Cappellii C, Stravato VM, Rotino GL, Buonaurio R Sources of resistance among *Solanum* spp. to an Italian isolate of *Fusarium oxysporum* f sp. *Melongenae*.

[7] Collonnier C, Mulya K, Fock I (2001). Source of resistance against *Ralstonia solanacearum* in fertile somatic hybrids of eggplant (*Solanum melongena* L.) with *Solanum aethiopicum* L.. *Plant Science*.

[8] Ano G, Hebert Y, Prior P, Messiaen C (1991). A new source of resistance to bacterial wilt of eggplants obtained from a cross: *Solanum aethiopicum* L × *Solatium melongena* L. *Agronomie*.

[9] Daunay MC, Lester RN, Laterrot H, Hawkes JH, Lester RN, Nee M, Estrada N (1991). The use of wild species for the genetic improvement of brinjal eggplant (*Solanum melongena*) and tomato (*Lycopersicon esculentum*). *Solanaceae III: Taxonomy, Chemistry, Evolution*.

[10] Rizza F, Mennella G, Collonnier C (2002). Androgenic dihaploids from somatic hybrids between *Solanum melongena* and *S. aethiopicum* group *gilo* as a source of resistance to *Fusarium oxysporum* f. sp. *Melongenae*. *Plant Cell Reports*.

[11] Toppino L, Valè G, Rotino GL (2008). Inheritance of *Fusarium* wilt resistance introgressed from *Solanum aethiopicum* Gilo and Aculeatum groups into cultivated eggplant (*S. melongena*) and development of associated PCR-based markers. *Molecular Breeding*.

[12] Fukuhara K, Kubo I (1991). Isolation of steroidal glycoalkaloids from *Solanum incanum* by two countercurrent chromatographic methods. *Phytochemistry*.

[13] Lester RN (1986). Taxonomy of scarlet eggplants, *Solanum aethiopicum* L.. *Acta Horticulturae*.

[14] Sánchez-Mata M, Yokoyama WE, Hong Y, Prohens J (2010). *α*-solasonine and *α*-solamargine contents of gboma (*Solanum macrocarpon* L.) and scarlet (*Solanum aethiopicum* l.) eggplants. *Journal of Agricultural and Food Chemistry*.

[15] Oshiro N, Kuniyoshi K, Nakamura A, Araki Y, Tamanaha K, Inafuku Y (2008). A case of food poisoning due to ingestion of Eggplant, *Solanum melongena*, grafted on Devil’s trumpet, *Datura metel*. *Journal of the Food Hygienic Society of Japan*.

[16] Yasinok AE, Sahin FI, Eyidogan F, Kuru M, Haberal M (2009). Grafting tomato plant on tobacco plant and its effect on tomato plant yield and nicotine content. *Journal of the Science of Food and Agriculture*.

[17] Daunay MC, Chaput MH, Sihachakr D, Allot M, Vedel F, Ducreux G (1993). Production and characterization of fertile somatic hybrids of eggplant (*Solanum melongena* L.) with *Solanum aethiopicum* L.. *Theoretical and Applied Genetics*.

[18] Bhojwani SS, Razdan MK (1996). *Plant Tissue Culture: Theory and Practice, a Revised Edition*.

[19] Boscaiu M, Donat P, Llinares J, Vicente O (2012). Stress-tolerant wild plants: a source of knowledge and tools for the genetic improvement of stress tolerance in crop plants. *Notulae Botanicae Horti Agrobotanici Cluj-Napoca*.

[20] Geier T, Eimert K, Scherer R, Nickel C (2008). Production and rooting behaviour of rolB-transgenic plants of grape rootstock “Richter 110” (*Vitis berlandieri* × *V. rupestris*). *Plant Cell, Tissue and Organ Culture*.

[21] Han J, Park S, Shigaki T, Hirschi KD, Kim CK (2009). Improved watermelon quality using bottle gourd rootstock expressing a Ca^2+^/H^+^ antiporter. *Molecular Breeding*.

[22] Li Y, Zhang Y, Feng F (2010). Overexpression of a Malus vacuolar Na^+^/H^+^ antiporter gene (MdNHX1) in apple rootstock M.26 and its influence on salt tolerance. *Plant Cell, Tissue and Organ Culture*.

[23] Kamat MG, Rao PS (1978). Vegetative multiplication of eggplants (*Solanum melongena*) using tissue culture techniques. *Plant Science Letters*.

[24] Mukherjee SK, Rathinasabapathi B, Gupta N (1991). Low sugar and osmotic requirements for shoot regeneration from leaf pieces of *Solanum melongena* L.. *Plant Cell, Tissue and Organ Culture*.

[25] Sharma P, Rajam MV (1995). Genotype, explant and position effects on organogenesis and somatic embryogenesis in eggplant (*Solanum melongena* L.). *Journal of Experimental Botany*.

[26] Magioli C, Rocha APM, de Oliveira DE, Mansur E (1998). Efficient shoot organogenesis of eggplant (*Solanum melongena* L.) induced by thidiazuron. *Plant Cell Reports*.

[27] Franklin G, Sheeba CJ, Sita GL (2004). Regeneration of eggplant (*Solanum melongena* L.) from root explants. *In Vitro Cellular and Developmental Biology*.

[28] Xing Y, Yu Y, Luo X, Zhang J-N, Zhao B, Guo Y-D (2010). High efficiency organogenesis and analysis of genetic stability of the regenerants in *Solanum melongena*. *Biologia Plantarum*.

[29] Matsuoka H, Hinata K (1979). NAA-induced organogenesis and embryogenesis in hypocotyl callus of *Solarium melongena* L.. *Journal of Experimental Botany*.

[30] Gleddie S, Keller W, Setterfield G (1983). Somatic embryogenesis and plant regeneration from leaf explants and cell suspensions of *Solanum melongena* (eggplant). *Canadian Journal of Botany*.

[31] Acciarri N, Restaino F, Vitelli G (2002). Genetically modified parthenocarpic eggplants: improved fruit productivity under both greenhouse and open field cultivation. *BMC Biotechnology*.

[32] Prabhavathi V, Yadav JS, Kumar PA, Rajam MV (2002). Abiotic stress tolerance in transgenic eggplant (*Solanum melongena* L.) by introduction of bacterial mannitol phosphodehydrogenase gene. *Molecular Breeding*.

[33] Arpaia S, Mennella G, Onofaro V, Perri E, Sunseri F, Rotino GL (1997). Production of transgenic eggplant (*Solanum melongena* L.) resistant to Colorado potato beetle (*Leptinotarsa decemlineata* say). *Theoretical and Applied Genetics*.

[34] Pal JK, Singh M, Rai M, Satpathy S, Singh DV, Kumar S (2009). Development and bioassay of *CryiAc*-transgenic eggplant (*Solarium melongena* L.) resistant to shoot and fruit borer. *Journal of Horticultural Science and Biotechnology*.

[35] Gisbert C, Prohens J, Nuez F (2006). Efficient regeneration in two potential new crops for subtropical climates, the scarlet (*Solanum aethiopicum*) and gboma (*S. macrocarpon*) eggplants. *New Zealand Journal of Crop and Horticultural Science*.

[36] Murashige T, Skoog F (1962). A revised medium for rapid growth and bioassays with tobacco tissue cultures. *Physiologia Plantarum*.

[37] Shivaraj G, Rao S (2011). Rapid and efficient plant regeneration of eggplant (*Solanum melongena* L.) from cotyledonary leaf explants. *Indian Journal of Biotechnology*.

[38] Trujillo-Moya C, Gisbert C, Vilanova S, Nuez F (2011). Localization of QTLs for *in vitro* plant regeneration in tomato. *BMC Plant Biology*.

[39] Chitra DSV, Padmaja G (2005). Shoot regeneration via direct organogenesis from *in vitro* derived leaves of mulberry using thidiazuron and 6-benzylaminopurine. *Scientia Horticulturae*.

[40] Guo B, Abbasi BH, Zeb A, Xu LL, Wei YH (2011). Thidiazuron: a multi-dimensional plant growth regulator. *African Journal of Biotechnology*.

[41] Sun J, Lei PD, Zhang ZZ (2012). Shoot basal ends as novel explants for *in vitro* plantlet regeneration in an elite clone of tea. *Journal of Horticultural Science and Biotechnology*.

[42] Gupta S, Mahalaxmi V (2009). *In vitro* high frequency direct plant regeneration from whole leaves of blackberry. *Scientia Horticulturae*.

[43] Hou F, Sama EA, Hughes GH, Abbas MS, Shahba MA (2012). An efficient *in vitro* propagation protocol of cocoyam [*Xanthosoma sagittifolium* (L.) Schott. *The Scientific World Journal*.

[44] Coupe M, Dauzac J (1992). Effect of ethylene on enzymatic-activities involved in the browning of *Hevea brasiliensis* callus. *Physiologia Plantarum*.

[45] Wu LM, Wei YM, Zheng YL (2006). Effects of silver nitrate on the tissue culture of immature wheat embryos. *Russian Journal of Plant Physiology*.

[46] Kucharska D, Gruchala A, Orlikowska T (2006). *In vitro* propagation of four rose rootstocks. *Propagation of Ornamental Plants*.

